# Evolved resistance to colistin and its loss due to genetic reversion in *Pseudomonas aeruginosa*

**DOI:** 10.1038/srep25543

**Published:** 2016-05-06

**Authors:** Ji-Young Lee, Young Kyoung Park, Eun Seon Chung, In Young Na, Kwan Soo Ko

**Affiliations:** 1 Department of Molecular Cell Biology, Sungkyunkwan University School of Medicine, Suwon 16419, South Korea

## Abstract

The increased reliance on colistin for treating multidrug-resistant Gram-negative bacterial infections has resulted in the emergence of colistin-resistant *Pseudomonas aeruginosa*. We attempted to identify genetic contributors to colistin resistance *in vitro* evolved isogenic colistin-resistant and -susceptible strains of two *P. aeruginosa* lineages (P5 and P155). Their evolutionary paths to acquisition and loss of colistin resistance were also tracked. Comparative genomic analysis revealed 13 and five colistin resistance determinants in the P5 and P155 lineages, respectively. Lipid A in colistin-resistant mutants was modified through the addition of 4-amino-L-arabinose; this modification was absent in colistin-susceptible revertant strains. Many amino acid substitutions that emerged during the acquisition of colistin resistance were reversed in colistin-susceptible revertants. We demonstrated that evolved colistin resistance in *P. aeruginosa* was mediated by a complicated regulatory network that likely emerges through diverse genetic alterations. Colistin-resistant *P. aeruginosa* became susceptible to the colistin upon its withdrawal because of genetic reversion. The mechanisms through which *P. aeruginosa* acquires and loses colistin resistance have implications on the treatment options that can be applied against *P. aeruginosa* infections, with respect to improving bactericidal efficacy and preventing further resistance to antibiotics.

The emergence and dissemination of antibiotic-resistant bacteria has had a profound influence on human and animal health and welfare, along with having economic consequences^1^. Antibiotic resistance can be classified as intrinsic, acquired, or adaptive^2^. Intrinsic resistance is not related to antibiotic selection but to the specific features of the bacterium. For example, many Gram-negative bacteria are resistant to various antibiotics because they possess an outer membrane with low permeability that functions as an extra barrier, preventing the entry of antibiotics into the cell. In addition, many bacteria contain efflux systems that pump antibiotics out of the bacterial cell.

Acquired resistance is the result of a spontaneous genetic mutation, or the horizontal acquisition of resistance genes from other bacteria *via* conjugation, transduction, or transformation^3^. Acquired resistance provides a selective advantage in the presence of antibiotic compounds, and a gene encoding antibiotic resistance can readily spread through a bacterial ecosystem. In acquired resistance, once a bacterium becomes resistant to an antibiotic, it is unable to return to susceptibility^4^. Bacteria can also become resistant to antibiotics through the development of adaptive resistance. Adaptive resistance is defined as reduced antimicrobial killing in populations of bacteria that were originally susceptible to a particular antibiotic agent^5^,^6^. It involves a transient increase in the ability of bacteria to survive the antibiotic, mainly because of alterations in gene and/or protein expression levels triggered by environmental conditions such as stress, nutrient conditions, and sub-inhibitory levels of the antibiotic^7^,^8^,^9^. In contrast to intrinsic and acquired resistance mechanisms, which are stable and can be transmitted to progeny, adaptive resistance is transient and is usually lost upon removal of the antibiotic agent. This type of resistance has been reported for aminoglycosides and polymyxins (polymyxin B and colistin) in *Pseudomonas aeruginosa* and other Gram-negative bacilli^8^,^10^,^11^,^12^.

Adaptive resistance might be one of the reasons of the phenomenon that laboratory susceptibility results are not congruent with the clinical effectiveness of antibiotics. Polymyxin resistance in *P. aeruginosa* is known to be adaptive, which is characterized by induction of resistance in the presence of drug and reversal to the susceptible phenotype in its absence^8^. Although the experimental and clinical implications of adaptive resistance to polymyxins have yet to be seen, they are administered in larger initial and longer interval bolus doses, like aminoglycosides due to the adaptive resistance in addition to concentration-dependent killing and post-antibiotic effect^8^,^10^. Thus, an understanding of the mechanisms behind evolution of resistance will assist in determining the optimal way to employ polymyxins and help in the development of new compounds that do not result in resistance.

Polymyxins bind to lipopolysaccharide (LPS), the major constituent of the outer membrane in Gram-negative bacteria, through interactions with phosphates and fatty acids of LPS core and lipid A moieties^13^,^14^. These interactions subsequently result in cell lysis and death^15^. A recent rise in infections caused by multidrug-resistant (MDR) Gram-negative bacteria, especially those resistant to carbapenems, has led to the use polymyxins as a last resort treatment option^16^,^17^,^18^,^19^. Increased use of polymyxins in therapy has resulted in the evolution of bacterial isolates with reduced susceptibility to this class of antibiotics worldwide^20^,^21^,^22^.

Polymyxin resistance in *P. aeruginosa* and other Gram-negative bacteria is associated with the addition of 4-amino-L-arabinose (L-Ara4N) or phosphoethanolamine (pEtN) to lipid A and core oligosaccharide components. This results in a reduction of the net negative charge of the outer membrane^23^,^24^,^25^. The regulatory two-component systems (TCSs) PhoP-PhoQ (PhoPQ) and PmrA-PmrB (PmrAB) play important roles in lipid A modification, which subsequently results in *P. aeruginosa* becoming resistant to polymyxins^26^. Amino acid alterations in PhoPQ and PmrAB are associated with polymyxin resistance in clinical *P. aeruginosa* isolates^27^,^28^,^29^,^30^. The ParRS and CprRS TCSs were recently found to be involved in *P. aeruginosa* polymyxin resistance^12^,^31^. The precise molecular details of these resistance mechanisms remain unclear, although there is a correlation between polymyxin susceptibility and alterations in these TCSs. In a previous study examining the evolution of polymyxin resistance in *P. aeruginosa* mutants, it was found that individual TCSs (PmrAB, PhoPQ, ParRS, and CprRS) were not essential for the acquisition of colistin resistance^32^. Therefore, it is possible that alternative or compensatory pathways exist and these might be involved in the evolution of colistin resistance^32^,^33^.

Although several mechanisms on the evolution of colistin-resistance have been known as above, there are relatively limited data to explain the reversion of colistin-resistant *P. aeruginosa* mutants to susceptible phenotype in the absence of antibiotics. In the current study, we obtained colistin-resistant mutants and colistin-susceptible revertants from two clinical colistin-susceptible wild-type *P. aeruginosa* strains (P5 and P155), and performed a comparative genomic analysis. We identified genetic factors involved in the acquisition and reversal of colistin resistance in *P. aeruginosa*. In addition, we found that evolved resistance to colistin was reversed upon withdrawal of the antibiotic and likely occurred due to genetic reversions.

## Results

### Acquisition and loss of colistin resistance

We previously obtained *in vitro*-selected colistin-resistant mutants from colistin-susceptible *P. aeruginosa* parental strains, P5 and P155, through repeated exposure to sub-inhibitory concentrations of colistin^30^. The process of selection of colistin-resistant mutants has been repeated severally and similar results could be obtained: colistin MICs increased abruptly after the exposure of 4 mg/L colistin. Resistant phenotypes were completely reversed and became susceptible to colistin by serial passaging in colistin-free medium. To track changes in colistin susceptibility during evolution of resistance and subsequent reversion in two *P. aeruginosa* strains, we determined colistin MICs for all strains.

**Figure 1:**
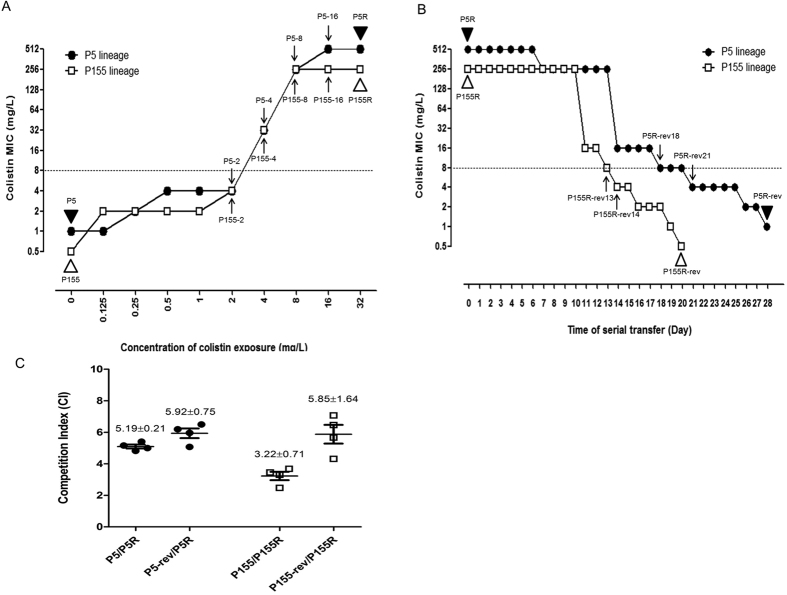
Changes in colistin minimum inhibitory concentration (MIC) during evolution of colistin resistance (**A**) and its loss (**B**) in *P. aeruginosa* P5 and P155 lineages. The points at which whole-genome sequencing WT strains and mutants of P5 and P155 lineages was conducted are indicated by the black and white triangles (▴ and ▵), respectively. Intermediate-stage mutants derived from P5 and P155 and subjected to sequence analysis for genes with alterations are indicated by arrows (↑ and ↓). (**C**) Competitive index (CI) as determined by competition assays. The CI represents the ratio of colony-forming units (CFUs) of a colistin-susceptible strain to the CFUs of a colistin-resistant strain (P5:P5R, P5R-rev:P5R, P155:P155R, and P155R-rev:P155R).

The P5 and P155 strains exhibited a colistin-resistant phenotype after serial passage for 6 days of exposure to 4 mg/L colistin (Fig. 1A). P5-4 and P155-4, intermediate strains selected in media with 4 mg/L colistin, showed markedly elevated colistin MICs (both, 32 mg/L), compared with intermediate strains selected in media with 2 mg/L colistin. After exposure to 32 mg/L colistin, viable cells (P5R and P155R) exhibited high levels of resistance, with colistin MICs of ≥256 mg/L. These resistant phenotypes were serially passaged in colistin-free medium to yield colistin-susceptible phenotypes (Fig. 1B). The colistin-susceptible revertants, P5R-rev and P155R-rev, which exhibited the same levels of susceptibility to colistin as their parental strains, were obtained after 28 and 20 passages, respectively, in media lacking antibiotics.

*In vitro* competition assays revealed that the competitive indices (CIs) of WT colistin-susceptible strains were higher than those for colistin-resistant mutants (5.19 ± 0.21 and 3.22 ± 0.71 for P5/P5R and P155/P155R, respectively) (Fig. 1C). These results imply that colistin-susceptible strains have higher fitness than do colistin-resistant mutants when incubated together. Our CI values indicate that fitness was restored in colistin-susceptible revertant strains (5.92 ± 0.75 and 5.85 ± 1.64 for P5R-rev/P5R and P155R-rev/P155R, respectively). However, it seems that these fitness advantages of the colistin-susceptible strains demonstrated by competition assays is not due to their growth rates (Fig. S1).

### Lipid A modifications in colistin-resistant mutants were restored in colistin-susceptible revertants

**Figure 2:**
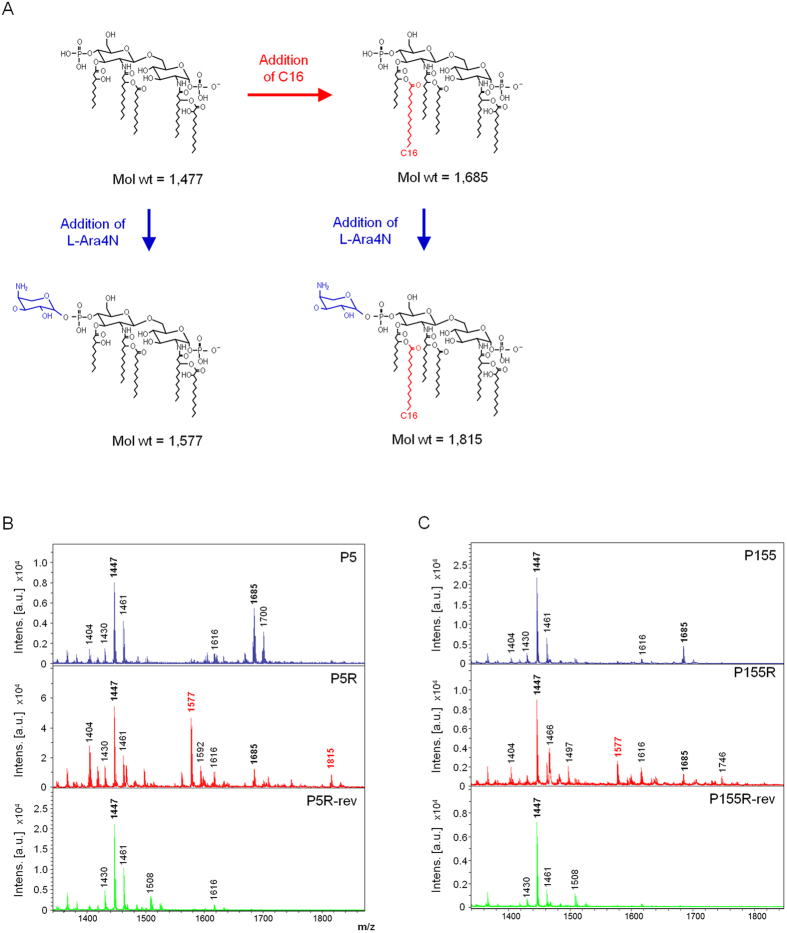
Lipid A structures and mass spectrometry analysis. (**A**) Lipid A structural modifications in colistin-susceptible *P. aeruginosa* P5 and P155, and *in vitro*-selected mutants. (**B**,**C**) MALDI-TOF mass spectrometry spectra for P5 and P155 isogenic stains, respectively.

To assess conformational changes in lipid A structure, we characterized and compared lipid A in all strains (P5, P155, P5R, P155R, P5R-rev, and P155R-rev) by using MALDI-TOF mass spectrometry. Lipid A from P5 exhibited major peaks at mass/charge (*m/z*) ratios of 1,447 and 1,685 (Fig. 2B), corresponding to a penta-acylated lipid A species and the addition of palmitate (C16:0), respectively (Fig. 2A). Lipid A from P5R had two major peaks at *m/z* 1,447 and 1,577; the size of the second peak indicated the addition of a L-Ara4N residue (Δ*m/z* = +130) to the penta-acylated species. There was also a minor peak at *m/z* 1,815, corresponding to the addition of an L-Ara4N residue and palmitate to the penta-acylated species. The mass spectrum for lipid A from P5R-rev was similar to that for WT P5, except that no peak was seen at *m/z* 1,685. This suggested the absence of palmitate. Additionally, there were no peaks at *m/z* 1,577 and *m/z* 1,815, indicating that P5R-rev lacked the aminoarabinosylated form observed in P5R.

For P155, the mass spectrum for lipid A was similar to that for P5, with a major peak at *m/z* 1,447 and a minor peak at *m/z* 1,685, corresponding to penta-acylated lipid A without and with palmitate, respectively (Fig. 2C). An additional peak at *m/z* 1,577 was found in lipid A of P155R, indicating aminoarabinosylation of the penta-acylated species. Lipid A of P155R-rev had no peaks at *m/z* 1,577 and *m/z* 1,685, indicating that modifications with palmitate and L-Ara4N had been restored.

For both *P. aeruginosa* strains, modification of lipid A with L-Ara4N was observed in the colistin-resistant mutants P5R and P155R, indicating that this modification correlated with colistin resistance. However, these modifications were absent in the colistin-susceptible revertants P5R-rev and P155R-rev.

### Comparative genomic analysis of P5 and P155 and their *in vitro-*selected mutants

To establish a link between specific genetic mutations and the evolution of colistin resistance, whole-genome sequencing was performed on all for six strains (P5, P155, P5R, P155R, P5R-rev, and P155R-rev). For each strain, around 20–35 million reads were generated, with a read coverage of 228- to 390-fold achieved with the PAO1 reference genome. Colistin-susceptible WT P5 and P155 strains showed no amino acid mutations in PmrAB, PhoPQ, ParRS, and CprRS, which are known to be associated with colistin resistance. In addition, they did not possess resistance plasmids including *mcr-1*, which confers colistin resistance^34^. While the imipenem-resistant P5 strain possesses *bla*_IMP-6_ gene, no metallo-β-lactamase genes were found in the susceptible P155 strain.

We identified 89 genetic alterations (87 SNPs and two indels) in P5 lineage (Table S1). Of these alterations, 70 (68 SNPs and two indels) were identified in coding sequences (CDSs), while 19 SNPs were identified in intragenic regions. Of the 70 alterations in CDSs, 18 (16 SNPs and two indels) were expected to result in non-synonymous substitutions, and were distributed among 13 CDSs, including *phoQ* (Table 1). The genes habouring mutations were categorized according to their PseudoCAP functional class in Pseudomonas Genome Database (www.pseudomonas.com). Out of these 13 genes, six genes (PA0334, PA1028, PA1224, PA2760, PA4089 and PA4092) were related to transport and metabolism. PA0334 encodes a major facilitator superfamily (MFS) transporter, and PA1028 encodes a oxidoreductase and is involved in the oxidation-reduction process that results in the removal or addition of one or more electrons to or from a substance. PA1224, PA2760, PA4089, and PA4092 encodes a NAD(P)H dehydrogenase, a porin, a short-chain dehydrogenase involves in fatty acid biosynthesis, and a 4-hydroxyphenylacetate 3-monooxygenase, respectively. Another two genes (PA1180 and PA4108) were linked to signal transduction mechanisms. PA1180, as is well known, encodes a two component sensor PhoQ which is involved in colistin resistance and PA4108 encodes a cyclic di-GMP phosphodiesterase. PA4000 and PA4406 encoding a lipoprotein A 3-monooxygenase and a UDP-3-O-acyl-N-acetylglucosamine deacetylase, respectively, are related to cell membrane biogenesis. PA0992 encodes a fimbrial subunit CupC1 related to motility and attachment. The remaining two genes (PA0043 and PA0260) appeared to encode hypothetical proteins of unknown function. Some of the alterations in P5R, such as PA0043, PA0334, PA1180 (*phoQ*), PA1224, PA4000, PA4092, and PA4108, were preserved in P5R-rev (Table 1), while other alterations were reversed and were the same as those in the parental colistin-susceptible strain.

We identified 31 genetic alterations (29 SNPs and 1 indel) in P155R and P155-rev (Table S2). Among these, 22 alterations (21 SNPs and 1 indel) were found in CDSs, and nine alterations (8 SNPs and 1 indel) were in intragenic regions. Of the 22 alterations in CDSs, seven SNPs were expected to result in non-synonymous substitutions, and were distributed among five CDSs (Table 2). Of these five genes, PA4777 (*pmrB*) encodes the sensor kinase of the well-known PmrAB TCS and PA0470 (*fiuA*) and PA4514 (*piuA*) encode a ferrichrome receptor and an outer membrane receptor, respectively, which are involved in iron transport. PA2157 and PA5088 were thought to encode hypothetical proteins. For P155R-rev, the five genetic mutations found in P155R were absent. Unlike the P5 isogenic mutants, the mutations found in P155R were not apparent in P155R-rev; however, P155R-rev did exhibit two new amino acid alterations (Lys598Glu and Lys601Glu) in PA0470.

SIFT scores were calculated to identify deleterious mutations (SIFT score ≤ 0.05) in P5R and P155R. Three mutations (Val260Gly in PA1180 [*phoQ*], Val62Glu in PA4089, and Gly85Ser in PA4406 [*lpxC*]) among 13 genes in P5R, and two mutations (Ala76Asp in PA2157 and Leu167Pro in PA4777 [*pmrB*]) among five genes in P155R were predicted to be deleterious (Tables 1 and 2). The Val260Gly substitution in PhoQ of P5R was predicted to be deleterious to protein function given that its SIFT score was 0.03; however, this substitution was not reversed in P5R-rev and this revertant strain was susceptible to colistin. The other non-synonymous mutations, including Lys123Gln in PhoQ (PA1180), were identified in the induced colistin-resistant mutants, and were thought to be generated during colistin challenge.

### Evolutionary paths to the acquisition and loss of colistin resistance

**Figure 3:**
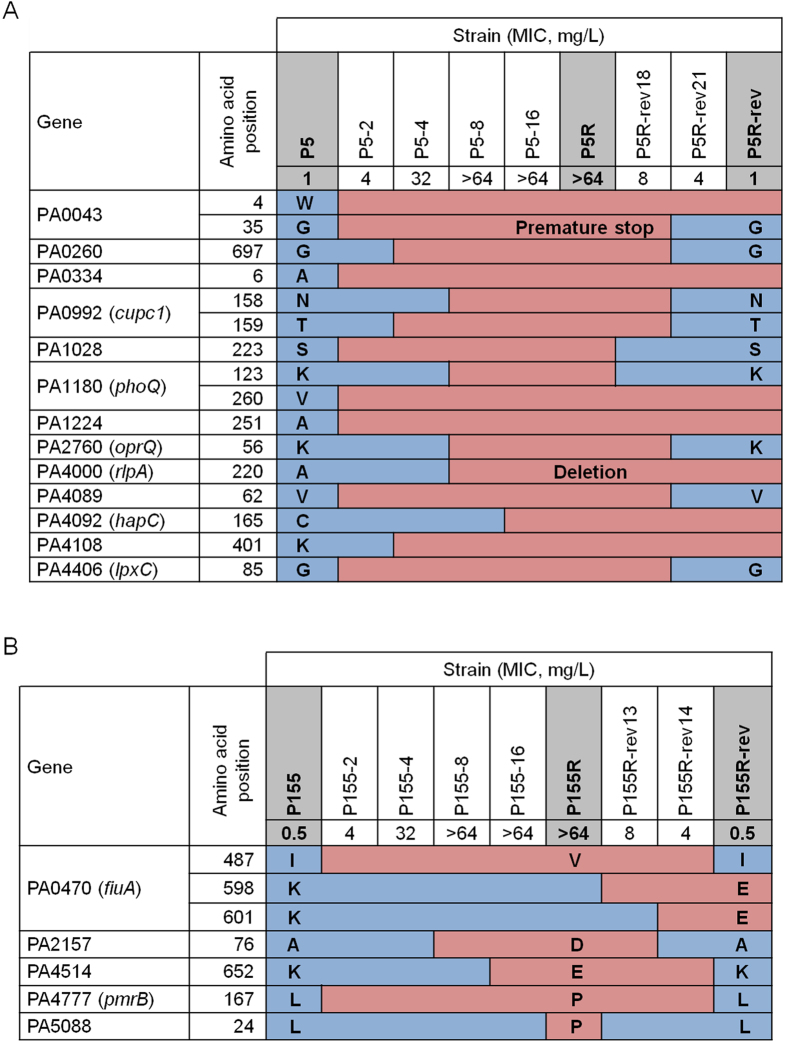
Amino acids substitution during the evolution and loss of colistin resistance in *P. aeruginosa* P5 **(A)** and P155 **(B).** The sequences of P5, P5R, P5R-rev, P155, P155R, and P155R-rev were determined by genome sequencing, while the sequences of all other strains were determined by the Sanger sequencing method.

During the emergence of antibiotic resistance in bacterial pathogens, three different scenarios can lead to loss of resistance: genetic reversion; compensation; or re-emergence^21^. We observed patterns of amino acid substitutions in intermediate-stage P5, P5R, and P5R-rev mutants (Fig. 3A). Of the 16 amino acid substitutions, seven were identified in both P5R and P5R-rev (Trp4Arg in PA0043, Ala6Thr in PA0334, Val260Gly in PA1180 [*phoQ*], Ala251Val in PA1224, Cys165Arg in PA4092 [*hapC*], Lys401Glu in PA4108, and the deletion of Ala220 in PA4000 [*rlpA*]). These emerged during the acquisition of colistin resistance and were preserved in the colistin-susceptible revertant strain. Another nine amino acid substitutions (Gly35Stop in PA0043, Gly697Ala in PA0260, Asn158Lys, and Thr159Ala in PA0992 [*cupc1*], Ser223Gly in PA1028, Lys123Gln in PA1180 [*phoQ*], Lys56Gln in PA2760 [*oprQ*], Val62Glu in PA4089, and Gly85Ser in PA4408 [*lpxC*]) that appeared as colistin resistance developed were absent in the colistin-susceptible revertant strain.

Five genes were mutated during the evolution of the P155R strain, with all of these mutations reversed in P155R-rev (Fig. 3B). Unlike those in the P5 isogenic strains, two additional alterations (Lys598Glu and Lys601Glu in PA0470) were observed in the intermediate-stage mutants as P155R developed into P155R-rev (P155R-rev13 and P155R-rev14). For both *P. aeruginosa* strains examined in this study, amino acid substitutions occurred at the various intermediate stages of evolution, and therefore were not always evident in our various analyses.

### Reversed mutations contribute to colistin resistance and susceptibility

To investigate the impact of specific mutations found in colistin-resistant mutants on colistin susceptibility, gene knock-out and complementation experiments were performed for three (PA0043, PA4089, and PA4406) and two (PA2157 and PA4777) genes in P5 and P155 lineages, respectively. These genes were selected based on the following criteria: (i) the mutations were predicted to be deleterious by SIFT scores and were found in colistin-resistant mutants, but not in colistin-susceptible strains or (ii) the mutations caused inactivation of the protein by frameshift mutation or premature stop.

**Figure 4:**
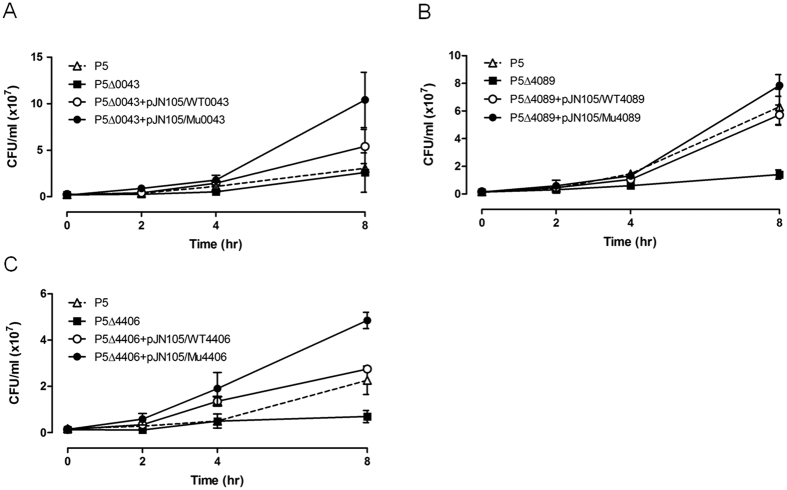
Time-kill assays for selected P. aeruginosa P5 mutants. Wild-type *P. aeruginosa* P5 (triangles), deletion mutants (squares), and complemented strains harbouring wild-type (unshaded circles) and mutant alleles (shaded circles, respectively) of PA0043 (**A**), PA4089 (**B**), and PA4406 (**C**), were grown in the presence of 0.5 mg/L colistin. Error bars were derived from three independent assays.

**Figure 5:**
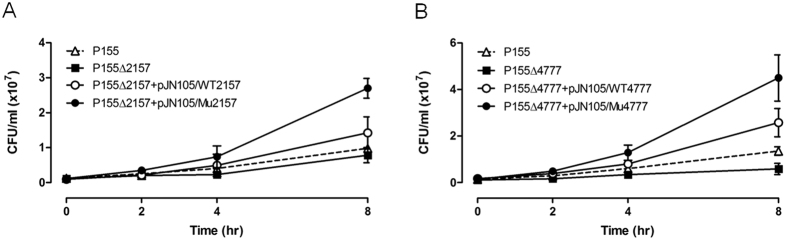
Time-kill assays for selected *P. aeruginosa* P155 mutants. Wild-type *P. aeruginosa* P155 (triangles), deletion mutants (squares), and complemented strains harbouring wild-type (unshaded circles) and mutant alleles (shaded circles) of PA2157 (**A**) and PA4777 (**B**), were grown in the presence of 0.25 mg/L colistin. Error bars were derived from three independent assays.

As minimum inhibitory concentration was not change greatly in deletion mutants and complemented strains, time-kill assays were performed with sub-inhibitory concentrations of colistin (0.5 and 0.25 mg/L for P5 and P155 mutants, respectively) for WT, deletion mutants, and complemented strains. All tested strains with alleles of resistant mutants, except for Val62Glu in PA4089 of P5, were more resistant to colistin compared with WT strains and deletion mutants (Figs 4 and 5). The complemented strains with WT alleles had their susceptibility to colistin restored to almost 100% (Figs 4 and 5). The PA4089 deletion in P5 (P5Δ4089) abrogated the killing effects of colistin compared with that in WT P5; however, complementation of WT or mutant alleles (P5Δ4089+pJN105/WT4089 and P5Δ4089+pJN105/Mu4089, respectively) did not result in a detectible difference in colistin susceptibility (Fig. 4B). The survival rates of deletion mutants (P5ΔPA4406 and P155ΔPA4777) of PA4406 (*lpxC*) in P5 and PA4777 (*pmrB*) in P155, which are involved in LPS biosynthesis and modification, were decreased >4-fold when colistin was present at sub-inhibitory concentrations, compared with those for their WT parents (Figs 4C and 5B). In addition, complemented strains with these mutant alleles (P5Δ4406+pJN105/Mu4406 and P155Δ4777+pJN105/Mu4777) exhibited a marked increase in colistin resistance after 8 h compared with that observed in complemented strains containing WT alleles. The mutations in these four genes that appeared in colistin-resistant mutants might be associated with the acquisition of colistin resistance. Reversions to the WT amino acid in the colistin-susceptible revertant strains might also be associated with restoration of colistin susceptibility.

## Discussion

In the current study, we obtained colistin-resistant mutants from two clinical colistin-susceptible wild-type *P. aeruginosa* strains (P5 and P155) through *in vitro* evolution of colistin resistance, and the colistin resistance developed by colistin pressure was reverted to susceptibility under environment without drug-sustaining effect. The susceptible strains acquired colistin resistance (MICs, from 4 mg/L to 32 mg/L) right after the exposure of 4 mg/L colistin. The development of colistin resistance seems to occur abruptly in particular concentration. However, the reversion to colistin susceptibility was not so fast. Although acquired colistin resistance may not be permanent, it was preserved for some time even in antibiotic-free media. Our evolved colistin resistance was regarded as adaptive resistance. Adaptive resistance is known as arising through alteration of target gene expression, but there is also a possibility that bacteria suffer mutations in various known or unknown related genes to alter target gene expression in the evolutionary process. In this study, we have discovered novel genetic pathways in *P. aeruginosa* that lead to colistin resistance and reversal to susceptibility by genetic reversion.

In *P. aeruginosa*, the evolution of colistin resistance is due to the modification of lipid A in LPS. This results in a reduction of the net charge of the outer membrane. This modification is known to be regulated by several TCSs, including PhoPQ, PmrAB, ParRS, and CprRS^12^,^29^,^31^. Loss of LPS due to mutations or disruption to genes involved in lipid A biosynthesis, such as *lpxA, lpxC*, and *lpxD*, has been reported for *A. baumannii*^35^,^36^. However, the involvement of other resistance mechanisms has also been proposed^32^.

Our comparative genomic analysis of the isogenic strains provided a comprehensive view regarding the evolution of colistin resistance in *P. aeruginosa*. Mutational trajectories resulting in colistin resistance were not limited to a few well-known resistance determinants. While *pmrAB, phoPQ* and *lpxC* are genes known to be related with colistin resistance in Gram-negative pathogens^28^,^30^,^37^,^38^, we associated PA0043, PA2157, and PA4089 with colistin resistance for the first time. In addition, none of the 13 known resistance determinants to colistin in P5R was apparent in P155R. The PA0470 (*fiuA*), PA2157, PA414 (*piuA*), PA4777 (*pmrB*), and PA5088 genes, which were associated with colistin resistance in P155R, were not identified in P5R. These findings suggest that the pathway to colistin resistance in *P. aeruginosa* varies among isolates. The P5R strain contained amino acid changes in numerous genes as compared with those in P155R, and colistin resistance of P5R was relatively stable after 20 days of passage without antibiotic selection pressure. Strain P155R, however, completely lost resistance to colistin within 20 days of serial passaging. The correlation between the number or type of genes with alterations and stability of colistin resistance is unclear at present.

One of the most interesting phenomena found in this study may be that the amino acid substitutions observed in colistin-resistant mutants were reversed for colistin-susceptible revertant strains. Sequence analysis ruled out the possibility that the colistin-susceptible revertants were a result of the re-emergence of susceptible parental strains. The alanine residue at position 290 of PA4000 (*rlpA*) was deleted in P5R, but was present in P5R-rev (Fig. 3A), indicating that the colistin-susceptible revertant was not a parental strain that had simply re-emerged. It is possible that the colistin-susceptible parental strains might be heterogeneous and could include colistin-resistant subpopulations, with the resistant subpopulations selected for in media containing colistin. However, this appears to be unlikely; population analysis profiling (data not shown) and patterns in amino acid changes failed to reveal any evidence of heteroresistance. In P155R-rev, additional mutations in the *fiuA* gene evolved during the restoration of colistin susceptibility, indicating that secondary mutations emerge and assist with restoring susceptibility to colistin (Fig. 3B). Although the genomic plasticity of *P. aeruginosa* is well-known^39^, this particular phenomenon has not been reported previously, as far as we know.

As is well known, altered expression of the *pmrH* gene is connected to the lipid A structure and this lipid A modification seems to play an important role in evolution of colistin resistance^30^. For both P5 and P155 *P. aeruginosa* lineages, modification of lipid A with L-Ara4N was observed in the colistin-resistant mutants P5R and P155R, and did not appear in lipid A of the revertant strains after the susceptibility resurgence (Fig. 2). These observations suggest that evolved colistin resistance in our study involves induced *pmrH* expression that increases the ability of survival in the presence of colistin, and was completely reverted to susceptible phenotype under environment without colistin-sustaining effect. Therefore, the evolved colistin resistance in our strains was regarded as adaptive resistance combined with genetic mutations. In addition to modification of lipid A with L-Ara4N, we also observed that the peak of lipid A structure with palmitate that seen in our parental strains and their resistant mutants disappeared in both revertant strains P5R-rev and P155R-rev (Fig. 2). Although the reason for disappearance of the palmitate peak in the revertant strains is not clear, it is speculated that the palmitoylated lipid A may not be associated with colistin resistance in our strains, as suggested by Gutu *et al.*^40^.

Based upon high mutant prevention concentrations of colistin in *P. aeruginosa* and other Gram-negative pathogens^41^, rapid evolution of colistin resistance is expected under antibiotic pressure. The use of colistin to treat infections by MDR Gram-negative bacteria has increased over time; however, levels of resistance to colistin are relatively low worldwide^42^,^43^. This could be due to the high fitness cost of colistin resistance, as we have shown (Fig. 1C). Genetic reversion of amino acids under antibiotic-free conditions might also explain the low levels of colistin resistance for *P. aeruginosa*. Adaptive resistance to aminoglycosides has been exhibited by *P. aeruginosa*; however, it remains unknown whether similar genetic reversions occur in response to this variety of antibiotics.

In conclusion, we have attempted to identify genetic factors involved in the evolution of colistin resistance in *P. aeruginosa*. In addition to well-known colistin resistance mechanisms, such as the PmrAB and PhoPQ TCSs and the LpxC mutations, we uncovered other genetic determinants that drive colistin resistance. Evolved resistance to colistin in our strains was reversed upon withdrawal of the antibiotic and likely occurred due to genetic reversion. The mechanisms through which *P. aeruginosa* acquires and loses colistin resistance have implications on the treatment options that can be applied against *P. aeruginosa* infections, with respect to improving bactericidal efficacy and preventing further resistance to antibiotics.

## Methods

### Bacterial strains and growth conditions

Two colistin-susceptible *P. aeruginosa* strains (P5 and P155) were isolated from patients with bacteraemia and used as parental strains^44^. Bacteria were grown in Luria-Bertani (LB) broth or LB agar plates, supplemented with the appropriate antibiotic at 37 °C. However, strains P5 and P155 harbouring plasmid pHK1014 were incubated at 30 °C. We used 50 mg/L gentamicin for *Escherichia coli* cultures, and 200 mg/L kanamycin and 50 mg/L gentamicin for *P. aeruginosa* cultures. All bacterial strains and plasmids used in this study are listed in Table S3.

### *In vitro* selection of induced colistin-resistant mutants and their revertant strains

From two parental strains P5 and P155, colistin-resistant mutants (P5R and P155R) were selected *in vitro* by serial passaging with progressively increasing concentrations of colistin^30^. Briefly, overnight cultures of colistin-susceptible strains in LB medium were diluted 1:100 into fresh medium containing subinhibitory concentrations of colistin increased serially (i.e., 0.125, 0.25, 0.5, 1, 2, 4, 8, and 16 mg/L) and incubated with vigorous shaking at 37 °C. In order to obtain single colistin-resistant population, spontaneous mutants grown in LB medium containing 16 mg/L of colistin were selected again on LB agar plates containing 32 mg/L colistin. Colistin MICs of individual colonies picked at random were confirmed by the broth microdilution method. As a result, two *in vitro*-selected colistin-resistant mutants, named P5R and P155R, were obtained. Their colistin MICs exceeded 64 mg/L. Aliquots of a culture inoculated with a single colony and grown overnight at 37 °C were stored in 20% glycerol at −80 °C and used to initiate reversion experiments.

We were then able to obtain the colistin-susceptible revertants P5R-rev and P155R-rev from P5R and P155R, respectively, by serial passaging in drug-free medium. Briefly, overnight cultures of the resistant mutants were diluted into fresh LB medium (1:1000) without colistin, and incubated with vigorous shaking at 37 °C for 24 h. To moniter changes in colistin susceptibility during reversion process from two colistin-resistant mutants, we determined colistin MICs for all passaging strains. The MICs of colistin were estimated using the broth microdilution method at the course of each transfer. After 28 serial passages of P5R and 20 passages of P155R, complete colistin-susceptible revertants, named P5R-rev and P155R-rev respectively, could be obtained. The colistin-susceptible revertants demonstrated colistin MICs of 0.5 or 1 mg/L.

### Determination of antibiotic susceptibility

The minimum inhibitory concentration (MIC) of colistin was determined according to the standard broth microdilution method, as described by the Clinical and Laboratory Standards Institute (CLSI) guidelines^45^. We assessed colistin concentrations ranging from 0.025 to 64 mg/L in cation-adjusted Mueller-Hinton broth (CA-MHB). The MIC was defined as the lowest concentration of antibiotic that yielded no visible growth of bacteria. We used *E. coli* ATCC 25922 and *P. aeruginosa* ATCC 27853 as control strains. MIC values were confirmed using three independent experiments.

### Competition assay

The fitness of colistin-resistant mutants and revertants was determined by competition assays. The relative fitness of colistin-susceptible wild-type (WT) strains and revertants against colistin-resistant mutants was determined by calculating an *in vitro* competitive index (CI), using a previously described method^46^,^47^ with some minor modifications. Overnight cultures of colistin-susceptible (WT or revertant) and colistin-resistant strains were inoculated to obtain a 0.5 McFarland standard and diluted 1:50 into 10 mL of LB broth with approximately 3 × 10^6^ CFU/ml of each strain. The diluted cultures were then mixed at a 1:1 (susceptible strain:resistant competitor) ratio and incubated at 37 °C for 20 h, with shaking at 180 rpm. The number of cells corresponding to each strain was determined by spreading serial 10-fold dilutions onto LB agar plates with or without 10 mg/L colistin. The CI was defined as the ratio of CFUs of colistin-susceptible bacteria to CFUs of colistin-resistant strain. Four independent competition experiments were performed to calculate median CI values.

### Lipid A isolation and structural analysis

Lipid A was extracted using the ammonium hydroxide-isobutyric acid method, as previously described^48^, then subjected to matrix-assisted laser desorption ionization-time of flight (MALDI-TOF) mass spectrometry. Washed cells (10 mg) were suspended in 400 μL of isobutyric acid:1 M ammonium hydroxide (5:3, vol/vol) and incubated at 100 °C for 2 h in a screw-cap test tube with occasional vortexing. The mixture was cooled on ice, centrifuged (5,000 rpm, 15 min), and the supernatant was transferred to a new tube and mixed with an equal volume of water. The mixture was lyophilized overnight at −70 °C, washed twice with 400 μL of methanol, and then centrifuged (5,000 rpm, 15 min). Insoluble lipid A was solubilized in 100 μL of chloroform:methanol:water (3:1.5:0.25, vol/vol/vol).

The structure of lipid A was analysed using MALDI-TOF mass spectrometry in negative-ion mode^49^,^50^. All MALDI-TOF analyses were performed on a Bruker Ultraflex III TOF/TOF mass spectrometer (Bruker Daltonics, Coventry, UK) using FlexControl 3.0 acquisition software. The matrix used for lipid A analysis was 2,5-dihydroxybenzoic acid (DHB; Sigma Chemical Co., St. Louis, MO). DHB solution (10 mg/mL) was prepared using a mixture of water:acetonitrile (1:4, vol/vol). All lipid A samples were mixed with DHB solution at a volume ratio of 1:1 and 1 μL of the resulting mixture was spotted on the MALDI metallic target. Lipid A from *E. coli* F583 (Sigma) was used as an external standard to calibrate mass.

### Whole-genome sequencing and confirmation of single nucleotide polymorphisms (SNPs) and indels

Whole-genome sequencing of P5, P155, P5R, P155R, P5R-rev, and P155R-rev was conducted at Macrogen Inc. (Seoul, Korea) with an Illumina HiSeq 2000 Sequencing System (Illumina, San Diego, CA, USA). Sequencing libraries were generated with a TruSeq DNA Sample Preparation Kit (Illumina), according to the manufacturer’s instructions. Paired-end read sequencing was performed with read lengths of 101 bp. Reads were trimmed and filtered based on quality score and then assembled onto the *P. aeruginosa* PAO1 reference genomic sequence (NC_002516.2) with BWA v0.5.9-r16. For detection of SNPs and short indels, SAM tools v0.1.14 and GATK v1.0.4937 were used. All non-synonymous mutations present in the *in vitro*-selected colistin-resistant mutants were confirmed by Sanger sequencing. For each mutation, we amplified a 500–800 bp PCR product containing the putative SNP and checked whether these mutations existed in the other strains we generated during *in vitro* induction of colistin resistance and reversion to colistin susceptibility. Primers were designed using Primer 3 software (http://frodo.wi.mit.ed/primer3/) and sequences were analysed using DNAStar software (DNAStar Inc., Madison, WI, USA).

To predict whether identified amino acid substitutions were likely to affect protein function, we used SIFT (http://sift.jcvi.org/). Based on amino acids appearing at each position, SIFT calculates the probability that an amino acid substitution at a position is tolerated, depending on the most frequent amino acid being tolerated. If the normalized value is less than 0.05, the substitution is predicted to be deleterious^51^,^52^.

### Generation of P5 and P155 deletion mutants

To generate deletion mutants of target genes in strains P5 and P155, an in-frame deletion was made by allelic exchange, as described previously, with some modifications^53^,^54^. Briefly, polymerase chain reactions (PCRs) were used to amplify two fragments (approximately 500 bp) flanking the coding region of the target gene. Segments of DNA containing the left and right regions of target genes were amplified from chromosomal DNA by using specific oligonucleotide primers (Table S4). Reverse primers for left fragments (L-R) and forward primers for right fragments (R-F) were designed to contain 20 nucleotides that overlapped with the 5′ and 3′ regions of the cassette to produce a fusion PCR amplicon. To replace the target gene and select for deletion mutants, a kanamycin resistance gene cassette (1,477 bp) amplified from pKD4 was used. Both fragments and the kanamycin resistance gene cassette were used as templates for another PCR. After both PCRs, the resulting fusion PCR products (>500 ng/μL) were transformed into competent cells harbouring the helper plasmid pHK1014. This particular plasmid contains the *aacC1* gene for the selection of transformants and the pUCP18 origin of replication^55^. The pHK1014 plasmid also has λRed recombinase functions for stimulation of homologous recombination at the target site of pKD46^54^. Homologous recombination between the fusion PCR products and the target gene in the chromosome was forced using chemical transformation^56^. Kanamycin-resistant transformants were selected on LB agar plates supplemented with 200 mg/L kanamycin. The replacement of target genes was confirmed by colony PCR. Cell lysates from mutant strains were used as PCR templates, along with primers L-F and R-R to verify the incorporation of replacement genes in the correct orientation. Correct incorporation of a fusion construct in a mutant strain generated PCR products (approximately 2.5 kb in size) that were larger or smaller than those from a WT strain.

### Complementation of target genes in deletion mutants

The open reading frame (ORF) of each gene and flanking DNA sequences were amplified by PCR using specific oligonucleotide primers (Table S4), and genomic DNA from colistin-susceptible parental strains and *in vitro*-selected colistin-resistant mutants as the template. Primers were engineered to carry the appropriate restriction sites for cloning and designed with Primer 3. Amplicons were digested with appropriate restriction enzymes and ligated into pJN105, which had been digested with the same restriction enzymes. For cloning, an aliquot of each ligation reaction was introduced into competent *E. coli* DH5α cells by using heat-shock treatment. Transformed bacteria were screened for recombinant plasmids, which were subsequently transformed into the deletion mutants by electroporation with a Gene Pulser (Bio-Rad). The transformation mixture was spread on LB agar plates containing 50 mg/L gentamicin and 50 mM L-arabinose. Gentamicin-resistant transformants were selected and presence of the target gene was confirmed by PCR analysis and sequencing.

### Quantitative *in vitro* bactericidal assays

Time-kill experiments were performed for WT P5 and P155, their deletion mutants, and complemented strains as described previously^57^, with some modifications. Because there were no differences between colistin MIC values obtained in CA-MH and LB broth, we used LB nutrient medium for time-kill assay to perform the experiment in the most basic and general environments. Bacteria were grown overnight in LB broth and then inoculated into LB broth at an initial concentration of 10^6^ CFU/mL. LB broth cultures contained 0.5 or 0.25 mg/L colistin, concentrations equivalent to 0.5 × MIC for P5 and P155, respectively. Cultures were incubated at 37 °C for 8 h with constant shaking (200 rpm). Aliquots were taken at designated time points (0, 2, 4, and 8 h post-inoculation), and serially diluted 10-fold in phosphate-buffered saline (PBS). Aliquots of each dilution (100 μL) were plated in duplicate on LB agar plates to determine the number of viable bacteria.

Changes in the colistin susceptibility of deletion mutants complemented with the WT or mutated allele were confirmed using a previously described quantitative bactericidal assay^25^,^58^. The various bacterial strains were grown and sub-cultured as described for the time-kill experiments, and then diluted in LB broth to 10^5^ CFU/mL. Cultures were exposed to 2-fold serial dilutions of colistin (1–16 mg/L or 0.5–8 mg/L) or a drug-free control for 30 min at 37 °C, and then spread on LB agar plates after a 10-fold dilution in PBS. Plates were incubated at 37 °C for 16–20 h to determine the number of surviving CFUs. All tests were repeated using three independent cultures.

## Additional Information

**How to cite this article**: Lee, J.-Y. *et al.* Evolved resistance to colistin and its loss due to genetic reversion in *Pseudomonas aeruginosa*. *Sci. Rep.* **6**, 25543; doi: 10.1038/srep25543 (2016).

## Supplementary Material

Supplementary Information

## Figures and Tables

**Table 1:** Non-synonymous mutations identified in P5R and P5R-rev.

No.	Locus ID	Gene name	Protein product	Genome position	Reference base (PAO1 and P5)	Altered base		Amino acid change		SIFT score
						P5R	P5R-rev	P5R	P5R-rev	
1	PA0043		Hypothetical protein	58499	G	–		Premature stop		ND
				58585	AA	GG	GG	Trp4Arg	Trp4Arg	0.33
2	PA0260		Hypothetical protein	291215	C	G		Gly697Ala		0.85
3	PA0334		Major facilitator superfamily (MFS) transporter	377174	C	T	T	Ala6Thr	Ala6Thr	0.42
4	PA0992	*cupC1*	Fimbrial subunit CupC1	1073758	T	G		Asn158Lys		0.93
				1073759	A	G		Thr159Ala		0.82
5	PA1028		Probable oxidoreductase	1115440	A	G		Ser223Gly		0.24
6	PA1180	*phoQ*	Two-component sensor PhoQ	1278728	A	C		Lys123Gln		0.29
				1279140	T	G	G	**Val260Gly**	**Val260Gly**	**0.03**
7	PA1224		Probable NAD(P)H dehydrogenase	1327775	C	T	T	Ala251Val	Ala251Val	0.74
8	PA2760	*oprQ*	Outer membrane porin	3120238	A	C		Lys56Gln		0.19
9	PA4000	*rlpA*	Lipoprotein A	4480573	AGC	–	–	Ala220*	Ala220*	ND
10	PA4089		Probable short-chain dehydrogenase	4572495	TC	AA		**Val62Glu**		**0.05**
11	PA4092	*hpaC*	4-Hydroxyphenylacetate 3-monooxygenase small subunit	4575824	T	C	C	Cys165Arg	Cys165Arg	0.10
12	PA4108		Cyclic di-GMP phosphodiesterase	4592387	A	G	G	Lys401Glu	Lys401Glu	0.56
13	PA4406	*lpxC*	UDP-3-O-acyl- N-acetylglucosamine deacetylase	4938935	C	T		**Gly85Ser**		**<0.01**

^*^Deleted amino acids.

Amino acid changes and SIFT scores in boldface type are substitutions predicted to affect the function of proteins. ND, not determined.

**Table 2:** Non-synonymous mutations identified in P155R and P155R-rev.

No.	Locus ID	Gene name	Protein product	Genome position	Reference base (PAO1 and P155)	Altered base		Amino acid change		SIFT score
						P155R	P155R-rev	P155R	P155R-rev	
1	PA0470	*fiuA*	Ferrichrome receptor FiuA	530979	T	C		Ile487Val		1.00
				530722	A		G		Lys598Glu	0.32
				530680	G		T		Lys601Glu	0.15
2	PA2157		Hypothetical protein	2377250	C	A		**Ala76Asp**		**<0.01**
3	PA4514		Probable outer membrane receptor for iron transport	5053924	T	C		Lys652Glu		0.73
4	PA4777	*pmrB*	Two-component regulator system signal sensor kinase PmrB	5365260	T	C		**Leu167Pro**		**<0.01**
5	PA5088		Hypothetical protein	5727168	A	G		Leu24Pro		0.14

Amino acid changes and SIFT scores in boldface type are substitutions predicted to affect the function of encoding proteins.
