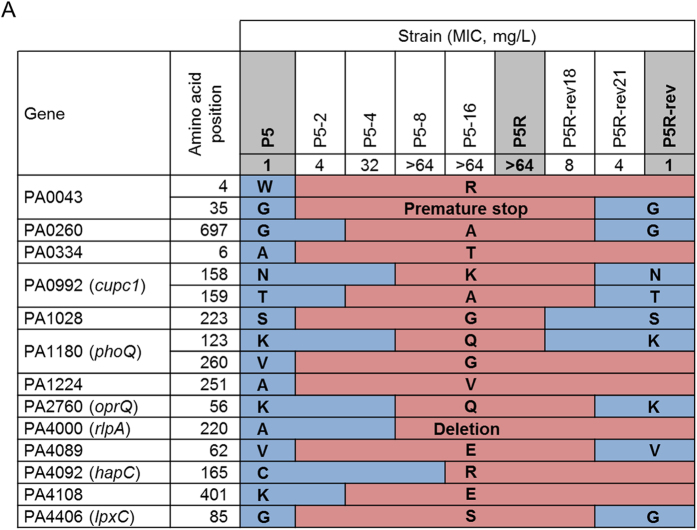# Corrigendum: Evolved resistance to colistin and its loss due to genetic reversion in *Pseudomonas aeruginosa*

**DOI:** 10.1038/srep30365

**Published:** 2016-07-25

**Authors:** Ji-Young Lee, Young Kyoung Park, Eun Seon Chung, In Young Na, Kwan Soo Ko

Scientific Reports 6: Article number: 2554310.1038/srep25543; published online: 05 06 2016; updated: 07 25 2016

In this Article, the amino acid sequences for strain P5R have been omitted from Figure 3A. The correct Figure 3A appears below as Figure 1.

## Figures and Tables

**Figure 1:**