# Supplementary material for: Evolved resistance to colistin and its loss due to genetic reversion in Pseudomonas aeruginosa
Source: Sci Rep. 2016 May 6;6:25543. doi: 10.1038/srep25543 (PMC4858706; doi:10.1038/srep25543)
Supplement: Supplementary Information [file srep25543-s1.doc]

**Evolved resistance to colistin and its loss due to genetic reversion in *Pseudomonas aeruginosa***

**Ji-Young Lee, Young Kyoung Park, Eun Seon Chung, In Young Na, & Kwan Soo Ko***

Department of Molecular Cell Biology, Samsung Biomedical Research Institute Sungkyunkwan University School of Medicine, Suwon 440-746, South Korea

**Supplementary Table S1**. Bacterial strains and plasmids used in this study

| Bacterial strain or plasmid | Genotype or characteristics | Reference |
| --- | --- | --- |
| **Strains** |  |  |
| ***P. aeruginosa*** |  |  |
| P5 | WT clinical isolate; Col-S | [1] |
| P5R | Induced colistin-resistant mutant, P5 background; Col-R | [2] |
| P5R-rev | Revertant derived from P5R; Col-S | [2] |
| P5R-rev18 | Population obtained from 18th passage of P5R cultures grown in colistin-free medium; Col-R | [2] |
| P5R-rev21 | Population obtained from 21th passage of P5R cultures grown in colistin-free medium; Col-I | [2] |
| P155 | WT clinical isolate; Col-S | [1] |
| P155R | Induced Col-R mutant, P155 background; Col-R | [2] |
| P155R-rev | Revertant derived from P155R; Col-S | [2] |
| P155R-rev13 | Population obtained from 13th passage of P155R cultures grown in colistin-free medium; Col-R | [2] |
| P155R-rev14 | Population obtained from 14th passage of P155R cultures grown in colistin-free medium; Col-I | [2] |
| P5Δ0043 | PA0043:: *aphIII*, P5 background; Km^r^ | This study |
| P5Δ0043-C1 | P5Δ0043 complemented with PA0043 from WT P5; Km^r^, Gm^r^ | This study |
| P5Δ0043-C2 | P5Δ0043 complemented with PA0043^Arg32Leu^ from P5R; Km^r^, Gm^r^ | This study |
| P5Δ4089 | PA4089:: *aphIII*, P5 background; Km^r^ | This study |
| P5Δ4089-C1 | P5Δ4089 complemented with PA4089 from WT P5; Km^r^, Gm^r^ | This study |
| P5Δ4089-C2 | P5Δ4089 complemented with PA4089^Val62Glu^ from P5R; Km^r^, Gm^r^ | This study |
| P5Δ4406 | PA4406:: *aphIII*, P5 background; Km^r^ | This study |
| P5Δ4406-C1 | P5Δ4406 complemented with PA4406 from WT P5; Km^r^, Gm^r^ | This study |
| P5Δ4406-C2 | P5Δ4406 complemented with PA4406^Gly85Ser^ from P5R; Km^r^, Gm^r^ | This study |
| P155Δ2157 | PA2157:: *aphIII*, P155 background; Km^r^ | This study |
| P155Δ2157-C1 | P155Δ2157 complemented with PA2157 from WT P155; Km^r^, Gm^r^ | This study |
| P155Δ2157-C2 | P155Δ2157 complemented with PA2157^Ala76Asp^ from P155R; Km^r^, Gm^r^ | This study |
| P155Δ4777 | PA4777:: *aphIII*, P155 background; Km^r^ | This study |
| P155Δ4777-C1 | P155Δ4777 complemented with PA4777 from WT P155; Km^r^, Gm^r^ | This study |
| P155Δ4777-C2 | P155Δ4777 complemented with PA4777^Leu167Pro^ from P155R; Km^r^, Gm^r^ | This study |
| ***E. coli*** |  |  |
| DH5α | F^–^ Φ80*lacZ*ΔM15 Δ(*lacZYA*-*argF*) *U169 recA1* *endA1 hsdR17* (r_K_^–^, m_K_^+^) *phoA* *supE44* λ^–^ *thi-1* *gyrA96 relA1* | [3] |
| **Plasmids** |  |  |
| pJN105 | *araC*-P_araBAD_ (broad-host-range vector); Gm^r^ | [4] |
| pUCP18 | *Escherichia*-*Pseudomonas* shuttle vectors; Amp^r^ | [5] |
| pKD46 | rep_pSC101_^ts^ *bla* P_araBAD_ *γ β* exo^+^; Amp^r^ | [6] |
| pHK1014 | pKD46 containing *aacC1* from pJN105 and RO1614*ori* from pUCP18; Amp^r^, Gm^r^ | [2] |
| pPA0043WT | PA0043 from WT P5 cloned into pJN105 at EcoRI/XbaI sites; Gm^r^ | This study |
| pPA0043Mu | PA0043^Arg32Leu^ from P5R cloned into pJN105 at EcoRI/XbaI sites; Gm^r^ | This study |
| pPA4089WT | PA4089 from WT P5 cloned into pJN105 at EcoRI/XbaI sites; Gm^r^ | This study |
| pPA4089Mu | PA4089^Val62Glu^ from P5R cloned into pJN105 at EcoRI/XbaI sites; Gm^r^ | This study |
| pPA4406WT | PA4406 from WT P5 cloned into pJN105 at EcoRI/XbaI sites; Gm^r^ | This study |
| pPA4406Mu | PA4406^Gly85Ser^ from P5R cloned into pJN105 at EcoRI/XbaI sites; Gm^r^ | This study |
| pPA2157WT | PA2157 from WT P155 cloned into pJN105 at EcoRI/XbaI sites; Gm^r^ | This study |
| pPA2157Mu | PA2157^Ala76Asp^ from P155R cloned into pJN105 at EcoRI/XbaI sites; Gm^r^ | This study |
| pPA4777WT | PA4777 from WT P155 cloned into pJN105 at EcoRI/XbaI sites; Gm^r^ | This study |
| pPA4777Mu | PA4777^Leu167Pro^ from P155R cloned into pJN105 at EcoRI/XbaI sites; Gm^r^ | This study |

Col-R, colistin-resistant; Col-I, colistin-intermediate; Col-S, colistin-susceptible; Km^r^, kanamycin-resistant; Gm^r^, gentamicin-resistant; and Amp^r^, ampicillin-resistant

**References**

1. Lee, J. Y., Song, J. H. & Ko, K. S. Identification of nonclonal *Pseudomonas aeruginosa* isolates with reduced colistin susceptibility in Korea. *Microb. Drug Resist*. **17,** 299-304 (2011).
2. Lee, J. Y. *et al*. Development of colistin resistance in *pmrA-, phoP-, parR-* and *cprR*-inactivated mutants of *Pseudomonas aeruginosa*. *J. Antimicrob. Chemother*. **69,** 2966-2971 (2014).
3. Woodcock, D. M. *et al*. Quantitative evaluation of *Escherichia coli* host strains for tolerance to cytosine methylation in plasmid and phage recombinants. *Nucleic Acids Res*. **17,** 3469-3478 (1989).
4. Newman, J. R. & Fuqua, C. Broad-host-range expression vectors that carry the L-arabinose-inducible *Escherichia coli* *araBAD* promoter and the *araC* regulator. *Gene* **227,** 197-203 (1999).
5. Schweizer, H. P. *Escherichia-Pseudomonas* shuttle vectors derived from pUC18/19. *Gene* **97,** 109-121 (1991).
6. Datsenko, K. A. & Wanner, B. L. One-step inactivation of chromosomal genes in *Escherichia coli* K-12 using PCR products. *Proc. Natl. Acad. Sci. USA* **97,** 6640-6645 (2000).

**Supplementary Table S2.** Oligonucleotide primers used for sequencing, gene inactivation, and cloning experiments

| Primer name | Sequence (5′→3′) | Amplicon size (bp) | | Reference | |
| --- | --- | --- | --- | --- | --- |
| **Sequencing** | | | | | |
| 0043S-F | ACAGCGGACTTTCCCTACG | 504 | | This study | |
| 0043S-R | GAGGAGGAAGGTCATCAGCA |  | |  | |
| 4089S-F | GCACCTCTACCTGCTCAACC | 648 | | This study | |
| 4089S-R | GCGGATGAAACCGTTGAC |  | |  | |
| 4406S-F | CAAACAACGCACCTTGAAGA | 607 | | This study | |
| 4406S-R | GTTCTGCGAACGCAGGTACT |  | |  | |
| 2157S-F | GGATCAAGGTCGAACTGGTG | 1278 | | This study | |
| 2157S-R | ATGCTGTCGGCCAGATACTC |  | |  | |
| pmr-F3 | TGCTGTCGAGCCTCAACCTG | 658 | | [1] | |
| pmr-R3 | GCTCGATGTCCTTGTCGATG |  | |  | |
| **Allelic replacement** | | | | | |
| Kan-F | AACAGTGAATTGGAGTTCGTCTTGT | 907 | | [2] | |
| Kan-R | GCTTTTTAGACATCTAAATCTAGGTA |  | |  | |
| 0043-LF | CTCAACTCAGCCACGAAAGA | 403 | | This study | |
| 0043-LR | GACGAACTCCAATTCACTGTTCAGGCTCAACAACAGATCCA |  | |  | |
| 0043-RF | AGATTTAGATGTCTAAAAAGCGATCCTGCTGTTGGTGGAG | 498 | | This study | |
| 0043-RR | CAGAGACGCCTCAGTCCAA |  | |  | |
| 4089-LF | GCACCTCTACCTGCTCAACC | 411 | | This study | |
| 4089-LR | GACGAACTCCAATTCACTGTTGTGCAGGAGGATGTCCAAC |  | |  | |
| 4089-RF | AGATTTAGATGTCTAAAAAGCGACGGCATCACCGTCAAC | 431 | | This study | |
| 4089-RR | GGTTGGGGATTTCCGATACT |  | |  | |
| 4406-LF | GCAGGATGACCTGGATTACC | 404 | | This study | |
| 4406-LR | GACGAACTCCAATTCACTGTTGTGCTCCACCGTATCCACTT |  | |  | |
| 4406-RF | AGATTTAGATGTCTAAAAAGCATCTGCTCGGCAACAGTCTT | 425 | | This study | |
| 4406-RR | GCGAACTTAGCGAAAATCCTT |  | |  | |
| 2157-LF | AAGTACATTCCGCTGCTGCT | 476 | | This study | |
| 2157-LR | GACGAACTCCAATTCACTGTTACGCTGAAAACGAAGTCCTC |  | |  | |
| 2157-RF | AGATTTAGATGTCTAAAAAGCCATGGCGACAAGGAACTGTA | 420 | | This study | |
| 2157-RR | CCTGGAGGAACACCAGGTC |  | |  | |
| 4777-LF | GCAACCAACTGGAGCAGAG | 413 | | This study | |
| 4777-LR | GACGAACTCCAATTCACTGTTGTTCGATGATCTCGGTGATG |  | |  | |
| 4777-RF | AGATTTAGATGTCTAAAAAGCCGATCTTCACCCGCTTCTAC | 431 | | This study | |
| 4777-RR | CAGGGTATGCAGGTCGTTCT |  | |  | |
| **Cloning** | | | | | |
| 0043CE-F | CCAAGAATTCCTCAACTCAGCCACGAAAGA | 1800 | This study | |  |
| 0043CX-R | CCAATCTAGACAGAGACGCCTCAGTCCAA |  |  | |  |
| 4089CE-F | CCAAGAATTCGCACCTCTACCTGCTCAACC | 1107 | This study | | |
| 4089CX-R | CCAATCTAGAGGTTGGGGATTTCCGATACT |  |  | | |
| 4406CE-F | CCAAGAATTCGCAGGATGACCTGGATTACC | 1320 | This study | | |
| 4406CX-R | CCAATCTAGAGCGAACTTAGCGAAAATCCTT |  |  | | |
| 2157CE-F | CCAAGAATTCAAGTACATTCCGCTGCTGCT | 1240 | This study | | |
| 2157CX-R | CCAATCTAGAGAAACCCTTGTGGGTGTTCA |  |  | | |
| 4777CE-F | CCAAGAATTCGCAACCAACTGGAGCAGAG | 1824 | This study | | |
| 4777CX-R | CCAATCTAGACAGGGTATGCAGGTCGTTCT |  |  | | |

**References**

1. Lee, J. Y. & Ko, K. S. Mutations and expression of PmrAB and PhoPQ related with colistin resistance in *Pseudomonas aeruginosa* clinical isolates. *Dign. Microbiol. Infect. Dis*. **78,** 271-276 (2014).

2. Song, J. H. *et al.* Identification of essential genes in *Streptococcus pneumoniae* by allelic replacement mutagenesis. *Mol. Cells* **19,** 365-374 (2005).

**Supplementary Table S3. List of SNPs and Indels detected in *in vitro-*selected mutants of P5, P5R and P5R-rev, compared to genome sequences of PAO1 and P5.**

| **No.** | | **Genome position** | | **Referencial base** | **Altered base** | | | **Product** | **Start** | | **End** | **Strand** |
| --- | --- | --- | --- | --- | --- | --- | --- | --- | --- | --- | --- | --- |
|  |  |  |  |  | **P5** | **P5R** | **P5R-rv** |  |  |  |  |  |
| 1 | | 39171 | | G |  | T | T |  |  | |  |  |
| 2 | | 58499 | | G |  | - |  | hypothetical protein | 57215 | | 58594 | - |
| 3 | | 58585 | | A |  | G | G | hypothetical protein | 57215 | | 58594 | - |
| 4 | | 58586 | | A |  | G | G | hypothetical protein | 57215 | | 58594 | - |
| 5 | | 116507 | | G |  | C |  | hypothetical protein | 115299 | | 117521 | + |
| 6 | | 116738 | | C |  | A |  | hypothetical protein | 115299 | | 117521 | + |
| 7 | | 151882 | | G |  | A | A |  |  | |  |  |
| 8 | | 163189 | | G |  | A |  | hydroxydechloroatrazine ethylaminohydrolase | 161906 | | 163252 | + |
| 9 | | 271755 | | G |  | A | A | porin | 270576 | | 271838 | - |
| 10 | | 276534 | | C |  | T | T | shikimate 5-dehydrogenase | 276483 | | 277334 | - |
| 11 | | 283263 | | T |  | C | C | hypothetical protein | 282915 | | 283553 | - |
| 12 | | 291215 | | C |  | G |  | hypothetical protein | 291157 | | 293304 | - |
| 13 | | 304137 | | A |  | G | G |  |  | |  |  |
| 14 | | 359911 | | C |  | T | T | hypothetical protein | 358931 | | 359917 | + |
| 15 | | 377174 | | C |  | T | T | major facilitator superfamily (MFS) transporter | 375954 | | 377189 | - |
| 16 | | 404398 | | A |  | G |  | gamma-glutamyltranspeptidase | 404389 | | 406119 | - |
| 17 | | 456558 | | T |  | C |  | component of chemotactic signal transduction system | 454126 | | 461541 | + |
| 18 | | 462094 | | T |  | C |  | methylesterase | 461537 | | 462565 | + |
| 19 | | 486376 | | A |  | G | G | hypothetical protein | 484964 | | 487153 | + |
| 20 | | 515770 | | C |  | G |  | hypothetical protein | 515656 | | 516027 | - |
| 21 | | 564596 | | C |  | T | T |  |  | |  |  |
| 22 | | 654164 | | G |  | A | A | organic solvent tolerance protein OstA | 653756 | | 656527 | - |
| 23 | | 811240 | | T |  | C |  |  |  | |  |  |
| 24 | | 955450 | | C |  | G | G |  |  | |  |  |
| 25 | | 1029251 | | C |  | T | T | hypothetical protein | 1028172 | | 1029497 | + |
| 26 | | 1070003 | | A |  | C | C | hypothetical protein | 1069769 | | 1070170 | + |
| 27 | | 1029251 | | C |  | T | T | hypothetical protein | 1028172 | | 1029497 | + |
| 28 | | 1070003 | | A |  | C | C | hypothetical protein | 1069769 | | 1070170 | + |
| 29 | | 1073758 | | T |  | G |  | fimbrial subunit CupC1 | 1073285 | | 1073899 | + |
| 30 | | 1073759 | | A |  | G |  | fimbrial subunit CupC1 | 1073285 | | 1073899 | + |
| 31 | | 1093664 | | C |  | T | T | dihydrodipicolinate synthase | 1093251 | | 1094126 | + |
| 32 | | 1115440 | | A |  | G |  | oxidoreductase | 1114774 | | 1116057 | + |
| 33 | | 1124824 | | G |  | A | A |  |  | |  |  |
| 34 | | 1173512 | | T |  | G | G | flagellar hook-associated protein FlgK | 1172163 | | 1174211 | + |
| **Supplementary Table S3. (continued)** | | | | | | | | | | | | |
| **No.** | **Genome position** | | **Referencial**  **base** | | **Altered base** | | | **Product** | | **Start** | **End** | **Strand** |
|  |  |  |  |  | **P5** | **P5R** | **P5R-rv** |  |  |  |  |  |
| 35 | 1173515 | | C | |  | T | T | flagellar hook-associated protein FlgK | | 1172163 | 1174211 | + |
| 36 | 1178728 | | A | |  | C |  | two-component sensor PhoQ | | 1278362 | 1279705 | + |
| 37 | 1279140 | | T | |  | G | G | two-component sensor PhoQ | | 1278362 | 1279705 | + |
| 38 | 1327775 | | C | |  | T | T | NAD(P)H dehydrogenase | | 1327024 | 1327800 | + |
| 39 | 1383159 | | C | |  | T | T | tonB-dependent receptor | | 1381804 | 1383651 | + |
| 40 | 1471055 | | G | |  | C | C | transcriptional regulator | | 1471019 | 1471672 | - |
| 41 | 1471064 | | G | |  | A | A | transcriptional regulator | | 1471019 | 1471672 | - |
| 42 | 1665959 | | A | |  | G | G |  | |  |  |  |
| 43 | 1706169 | | A | |  | G | G |  | |  |  |  |
| 44 | 1714706 | | T | |  | C |  |  | |  |  |  |
| 45 | 2041498 | | C | |  | A | A | hypothetical protein | | 2036441 | 2043844 | + |
| 46 | 2041501 | | C | |  | G | G | hypothetical protein | | 2036441 | 2043844 | + |
| 47 | 2344239 | | T | |  | C |  | usher CupA3 | | 2343862 | 2346477 | + |
| 48 | 2344242 | | A | |  | G |  | usher CupA3 | | 2343862 | 2346477 | + |
| 49 | 2374704 | | A | |  | G | G | cardiolipin synthase 2 | | 2374605 | 2375807 | - |
| 50 | 2434629 | | C | |  | T | T | major facilitator superfamily (MFS) transporter | | 2433748 | 2435067 | + |
| 51 | 2541916 | | A | |  | G |  | AmbB | | 2541200 | 2544946 | - |
| 52 | 2541923 | | A | |  | C |  | AmbB | | 2541200 | 2544946 | - |
| 53 | 2558743 | | G | |  | A |  |  | |  |  |  |
| 54 | 2558857 | | C | |  | T | T |  | |  |  |  |
| 55 | 2647478 | | C | |  | A |  | PvdP | | 2646779 | 2648410 | - |
| 56 | 2647481 | | A | |  | G |  | PvdP | | 2646779 | 2648410 | - |
| 57 | 2770468 | | T | |  | G |  | hypothetical protein | | 2761924 | 2778804 | - |
| 58 | 3120238 | | A | |  | C |  | outer membrane protein | | 3120073 | 3121347 | + |
| 59 | 3146526 | | C | |  | G | G | hypothetical protein | | 3146251 | 3147327 | + |
| 60 | 3161774 | | T | |  | C | C | hypothetical protein | | 3161602 | 3162216 | - |
| 61 | 3251616 | | G | |  | A | A |  | |  |  |  |
| 62 | 3307056 | | C | |  | T | T |  | |  |  |  |
| 63 | 3352397 | | G | |  | A | A | NADH-quinone reductase subunit F | | 3351659 | 3352879 | - |
| 64 | 3526637 | | G | |  | A |  | nucleotide sugar epimerase/dehydratase WbpM | | 3524684 | 3526678 | - |
| 65 | 3526640 | | C | |  | A |  | nucleotide sugar epimerase/dehydratase WbpM | | 3524684 | 3526678 | - |
| 66 | 3684535 | | G | |  | C | C | hypothetical protein | | 3684166 | 3684717 | - |
| 67 | 3684538 | | A | |  | G | G | hypothetical protein | | 3684166 | 3684717 | - |
| 68 | 3772781 | | C | |  | G | G |  | |  |  |  |
| 69 | 4060086 | | G | |  | A | A | protein-L-isoaspartate O-methyltransferase | | 4059960 | 4060592 | - |
| **Supplementary Table S3. (continued)** | | | | | | | | | | | | |
| **No.** | **Genome position** | | **Referencial base** | | **Altered base** | | | **Product** | | **Start** | **End** | **Strand** |
|  |  |  |  |  | **P5** | **P5R** | **P5R-rv** |  |  |  |  |  |
| 70 | 4060098 | | C | |  | G | G | protein-L-isoaspartate O-methyltransferase | | 4059960 | 4060592 | - |
| 71 | 4348045 | | G | |  | T | T |  | |  |  |  |
| 72 | 4480573 | | GAG | |  | - |  | hypothetical protein | | 4480208 | 4481233 | - |
| 73 | 4572495 | | T | |  | A | A | 3-ketoacyl-ACP reductase | | 4572311 | 4573069 | + |
| 74 | 4572496 | | C | |  | A | A | 3-ketoacyl-ACP reductase | | 4572311 | 4573069 | + |
| 75 | 4575824 | | T | |  | C | C | 4-hydroxyphenylacetate 3-monooxygenase small subunit | | 4575332 | 4575841 | + |
| 76 | 4592387 | | A | |  | G | G | cyclic di-GMP phosphodiesterase | | 4591187 | 4592428 | + |
| 77 | 4682151 | | G | |  | A |  |  | |  |  |  |
| 78 | 4831149 | | A | |  | C | C | type IVb pilin%2C Flp | | 4830964 | 4831179 | + |
| 79 | 4832454 | | T | |  | C |  | chemotactic transducer PctC | | 4831372 | 4833267 | + |
| 80 | 4832457 | | C | |  | T |  | chemotactic transducer PctC | | 4831372 | 4833267 | + |
| 81 | 4938935 | | C | |  | T |  | UDP-3-O-[B3-hydroxymyristoyl] N-acetylglucosamine deacetylase | | 4938279 | 4939187 | - |
| 82 | 5100985 | | A | |  | C | C | type 4 fimbrial biogenesis protein PilY1 | | 5100683 | 5104165 | + |
| 83 | 5210639 | | G | |  | T | T |  | |  |  |  |
| 84 | 5256120 | | T | |  | C | C | hypothetical protein | | 5254738 | 5257581 | + |
| 85 | 5356253 | | T | |  | C |  | L-lactate permease | | 5355387 | 5357072 | + |
| 86 | 5506848 | | A | |  | G | G |  | |  |  |  |
| 87 | 5538897 | | A | |  | G |  | exoribonuclease RNase R | | 5538699 | 5541410 | - |
| 88 | 5701127 | | C | |  | T | T | polyhydroxyalkanoate synthesis protein PhaF | | 5700761 | 5701687 | - |
| 89 | 5707149 | | T | |  | C |  | sec-independent translocase | | 5706814 | 5707236 | + |

**Supplementary Table S4.** List of SNPs and Indels detected in *in vitro-*selected mutants of P155, P155R and P155R-rev, compared to genome sequences of PAO1 and P155.

| No. | **Genome position** | **Referential base** | **Altered base** | | | **Product** | **Start** | **End** | **Strand** |
| --- | --- | --- | --- | --- | --- | --- | --- | --- | --- |
|  |  |  | **P155** | **P155R** | **P155R-rv** |  |  |  |  |
| 1 | 54083 | G |  | A |  |  |  |  |  |
| 2 | 264613 | G |  | A |  |  |  |  |  |
| 3 | 398139 | G |  | - |  |  |  |  |  |
| 4 | 530680 | G |  |  | T | ferrichrome receptor FiuA | 530032 | 532437 | - |
| 5 | 530722 | A |  |  | G | ferrichrome receptor FiuA | 530032 | 532437 | - |
| 6 | 530979 | T |  | C |  | ferrichrome receptor FiuA | 530032 | 532437 | - |
| 7 | 789119 | T |  | A |  |  |  |  |  |
| 8 | 795457 | G |  | A |  | hypothetical protein | 794501 | 795790 | + |
| 9 | 896140 | T |  | C |  |  |  |  |  |
| 10 | 1302992 | G |  | A |  | hydrolase | 1302696 | 1303310 | + |
| 11 | 1535422 | C |  | T |  |  |  |  |  |
| 12 | 1650430 | A |  | C |  |  |  |  |  |
| 13 | 2376694 | - |  | GTCGCC |  | hypothetical protein | 2376541 | 2377476 | - |
| 14 | 2641529 | C |  | T |  | FpvR | 2641035 | 2642027 | + |
| 15 | 2647493 | A |  | G |  | PvdP | 2646779 | 2648410 | - |
| 16 | 2688198 | T |  | G |  | hypothetical protein | 2687497 | 2688705 | + |
| 17 | 2767579 | A |  | G |  | hypothetical protein | 2761924 | 2778804 | - |
| 18 | 2777875 | G |  | A |  | hypothetical protein | 2761924 | 2778804 | - |
| 19 | 3072067 | G |  | A |  |  |  |  |  |
| 20 | 5053924 | T |  | C |  | outer membrane receptor for iron transport | 5053619 | 5055877 | - |
| 21 | 5101441 | T |  | G |  | type 4 fimbrial biogenesis protein PilY1 | 5100683 | 5104165 | + |
| 22 | 5101447 | C |  | T |  | type 4 fimbrial biogenesis protein PilY1 | 5100683 | 5104165 | + |
| 23 | 5187917 | A |  | G |  | hypothetical protein | 5186414 | 5192875 | - |
| 24 | 5187923 | A |  | G |  | hypothetical protein | 5186414 | 5192875 | - |
| 25 | 5356253 | T |  | C |  | L-lactate permease | 5355387 | 5357072 | + |
| 26 | 5365261 | T |  | C |  | two-component regulator system signal sensor kinase PmrB | 5364760 | 5366193 | + |
| 27 | 5616711 | C |  | A |  | O-antigen ligase, WaaL | 5616310 | 5617512 | + |
| 28 | 5616717 | C |  | A |  | O-antigen ligase, WaaL | 5616310 | 5617512 | + |
| 29 | 5616720 | G |  | A |  | O-antigen ligase, WaaL | 5616310 | 5617512 | + |
| 30 | 5727168 | A |  | G |  | hypothetical protein | 5726360 | 5727238 | - |
| 31 | 5754296 | T |  | C |  |  |  |  |  |

**Supplementary Figure S1.** Bacterial growth curves for *P. aeruginosa* P5 (A) and P155 (B) lineages.

A

B
